# Supplementary material for: Identification of the Response-Related Biomarker of Bimonthly Hepatic Arterial Infusion Chemotherapy
Source: J Clin Med. 2021 Feb 7;10(4):629. doi: 10.3390/jcm10040629 (PMC7914951; doi:10.3390/jcm10040629)
Supplement: Supplementary file 1 [file jcm-10-00629-s001.pdf]

Supplementary Table S1

Profiles of HCC patients with liver cirrhosis in this study (n=96)

	Sorafenib (n=48)	B-HAIC (n=48)	P
Age (years, median)	73 (43-86)	70 (44-88)	N.S.
Sex (male/female)	40/8	38/10	N.S.
Etiology (HBV/HCV/Alcohol/Others)	8/33/3/4	10/33/4/1	N.S.
Child-Pugh classification (Class A/Class B)	48/0	22/26	p<0.01
Preceding treatments (yes/no)	43/5	47/1	N.S.
AFP (ng/mL) (range, median)	123 (3-293718)	57 (2.6-406875)	N.S.
DCP (mAU/mL) (range, median)	2483 (11-167252)	85 (8-268747)	N.S.
HCC numbers (1-3/4 and over)	12/36	12/36	N.S.
Intravascular invasion (with/without)	13/35	12/36	p<0.05
Extrahepatic metastasis (with/without)	22/26	2/46	p<0.01
HCC clinical stage (II/III/IV)	1/10/37	14/26/8	p<0.01
T factor (T2/T3/T4)	4/22/22	12/29/7	p<0.01

Categorical variables were tested with Chi-square test and continuous variables with Mann–Whitney U test.

HBV, hepatitis B virus; HCV, hepatitis C virus; HCC, hepatocellular carcinoma; AFP, alpha-fetoprotein; DCP, des-gamma-carboxy prothrombin; NS, not significant.

Supplementary Table S2

Profiles of HCC patients treated with B-HAIC (n=48)

	B-HAIC Child A (n=22)	B-HAIC Child B (n=26)	P
Age (years, median)	69 (44-88)	70 (56-82)	N.S.
Sex (male/female)	17/5	21/5	N.S.
Etiology (HBV/HCV/Alcohol/Others)	8/11/2/1	2/22/2/0	N.S.
Preceding treatments (yes/no)	22/0	25/1	N.S.
AFP (ng/mL) (range, median)	640 (2.6-406875)	57 (3.2-109267)	N.S.
DCP (mAU/mL) (range, median)	696 (10-268747)	85 (8-15459)	N.S.
HCC numbers (1-3/4 and over)	6/16	6/20	N.S.
Intravascular invasion (with/without)	10/12	2/24	p<0.01
Extrahepatic metastasis (with/without)	1/21	1/25	N.S.
HCC clinical stage (II/III/IV)	3/14/5	11/12/3	p<0.05
T factor (T2/T3/T4)	3/15/4	9/14/3	N.S.

Categorical variables were tested with Chi-square test and continuous variables with Mann–Whitney U test.

HBV, hepatitis B virus; HCV, hepatitis C virus; HCC, hepatocellular carcinoma; AFP, alpha-fetoprotein; DCP, des-gamma-carboxy prothrombin; NS, not significant.

Supplementary Table S3

Correlations between PFS and clinical data in patients treated with B-HAIC (n=48)

	Correlation coefficient	95% Confidence interval		P
		Lower limit	Upper limit-	
Albumin (g/dL)	0.1197	-0.1703	0.3905	0.4179
Total bilirubin (mg/dL)	-0.1605	-0.4252	0.1296	0.2759
Child-Pugh score (point)	-0.1254	-0.3954	0.1646	0.3959
AFP (ng/mL)	-0.2052	-0.4624	0.0838	0.1618
DCP (mAU/mL)	-0.1941	-0.4532	0.0953	0.1862

AFP, alpha fetoprotein; DCP, des-gamma-carboxy prothrombin; PFS, progression free survival; B-HAIC, Bimonthly hepatic arterial infusion chemotherapy

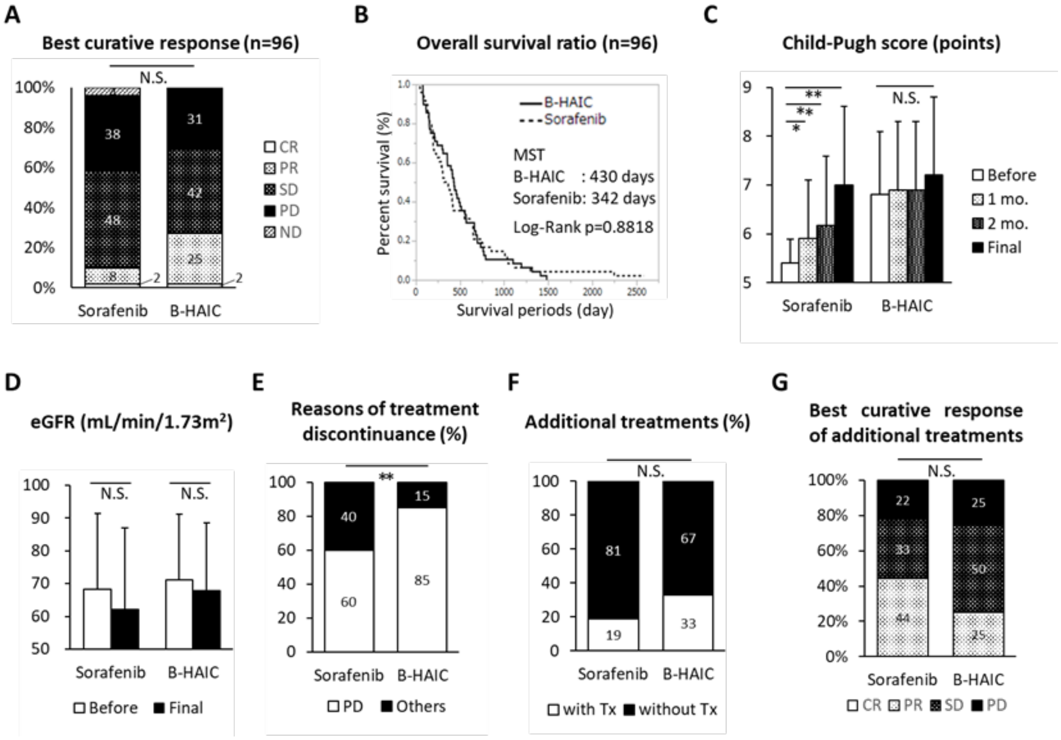

Supplementary Figure S1. Chemopreventive effects and adverse effects of sorafenib and B-HAIC on advanced HCC. (A) Patients treated with sorafenib were less likely to respond to treatment compared with those treated with B-HAIC (p = 0.065), although the disease control rates were similar between the groups. CR, complete response; PR, partial response; SD, stable disease; PD, progressive disease; ND, not determined. (B) Kaplan–Meier curves show the OS of patients in the B-HAIC group (solid black line, n = 48) and sorafenib group (dashed line, n = 48). (C, D) Changes in Child–Pugh scores and estimated glomerular filtration rate (eGFR) before and after the treatments in each group. The Child–Pugh score did not increase significantly during the B-HAIC treatment period in the B-HAIC groups, whereas it increased during the treatment period in the sorafenib group. (E) Most patients discontinued B-HAIC due to disease progression despite HFR, whereas a significantly larger population of patients in the sorafenib group had to discontinue treatment due to other reasons such as moderate-to-severe adverse effects (p = 0.0098). (F) Patients who were treated with B-HAIC exhibited a similar rate of additional chemotherapy to those treated with sorafenib, even though they had relatively poor HFR. (G) Best curative response of additional treatments. * p < 0.05, ** p < 0.01.

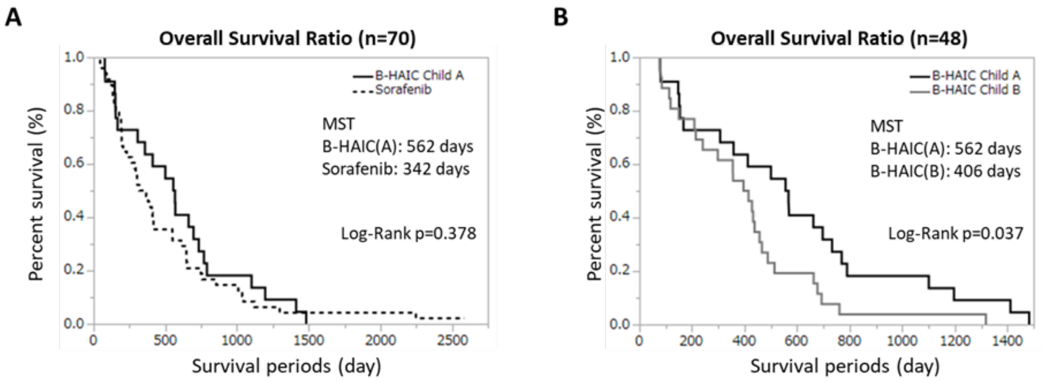

Supplementary Figure S2. Chemopreventive effects of sorafenib and B-HAIC on advanced HCC. (A) Kaplan–Meier curves show the OS of patients in the B-HAIC Child A group (solid black line, n = 22) and sorafenib group (dashed line, n = 48). No inferior outcome of B-HAIC to sorafenib treatment was indicated (p = 0.378), although some clinical background factors in each group were significantly different. (B) Kaplan–Meier curves showing the OS of patients in the B-HAIC Child A group (solid black line, n = 22) and B-HAIC Child B group (solid gray line, n = 26). A relatively superior outcome was seen the B-HAIC Child A group compared with the B-HAIC Child B group and showed a significant difference in the Log-Rank analysis (p = 0.037).

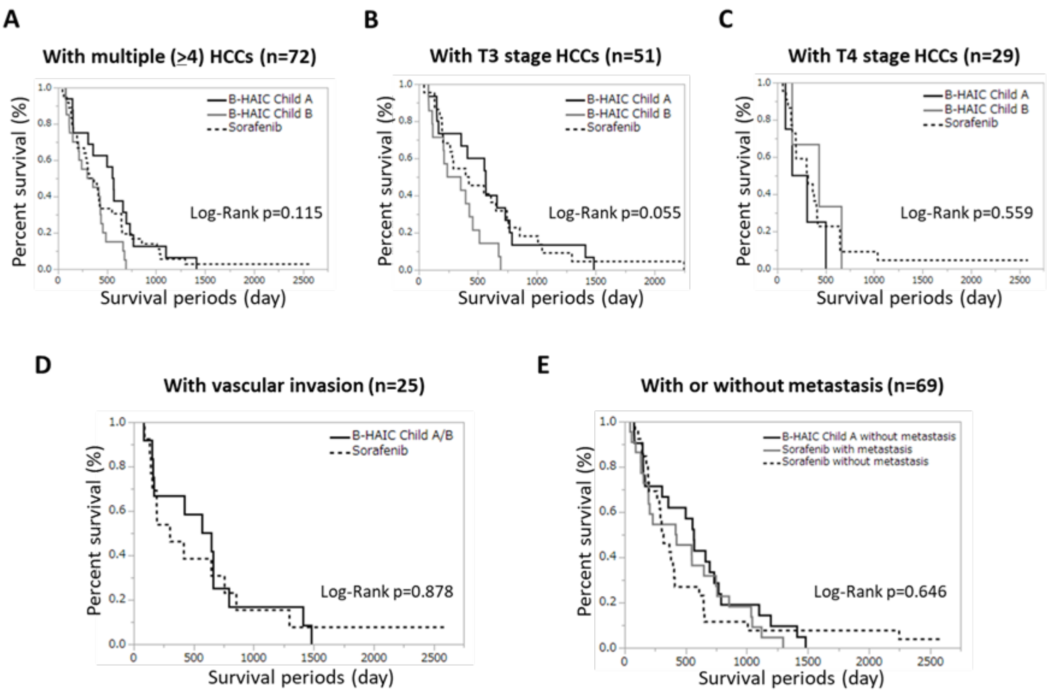

Supplementary Figure S3. Chemopreventive effects of sorafenib and B-HAIC on advanced HCC classified with HCC numbers, clinical tumor stage, intravascular invasion, and metastasis. (A) Kaplan–Meier curves show the OS of patients with four or more nodules of intrahepatic HCCs classified into the B-HAIC Child A group (solid black line, n = 16), B-HAIC Child B group (solid gray line, n = 20), and sorafenib group (dashed line, n = 36). (B) Kaplan–Meier curves show the OS of patients with T3 stage HCCs classified into the B-HAIC Child A group (solid black line, n = 15), B-HAIC Child B group (solid gray line, n = 14), and sorafenib group (dashed line, n = 22). (C) Kaplan–Meier curves show the OS of patients with T4 stage HCCs classified into the B-HAIC Child A group (solid black line, n = 4), B-HAIC Child B group (solid gray line, n = 3), and sorafenib group (dashed line, n = 22). (D) Kaplan–Meier curves of OS of patients with intravascular invasion treated with B-HAIC (solid black line, n = 12) and sorafenib (dashed line, n = 13). (E) Kaplan–Meier curves of OS of patients with or without extrahepatic metastasis classified into the B-HAIC Child A without metastasis group (solid black line, n = 21), sorafenib with metastasis group (solid gray line, n = 22), and sorafenib without metastasis group (dashed line, n = 26).
